# Psychological Distress and Coping Ability of Women at High Risk of Hereditary Breast and Ovarian Cancer before Undergoing Genetic Counseling—An Exploratory Study from Germany

**DOI:** 10.3390/ijerph18084338

**Published:** 2021-04-19

**Authors:** Beate Vajen, Magdalena Rosset, Hannah Wallaschek, Eva Baumann, Brigitte Schlegelberger

**Affiliations:** 1 Department of Human Genetics, Hanover Medical School, 30625 Hannover, Germany; Vajen.Beate@mh-hannover.de (B.V.); Wallaschek.Hannah@mh-hannover.de (H.W.); 2 Department of Journalism and Communication Research, Hanover University of Music, Drama and Media, 30175 Hannover, Germany; magdalena.rosset@ijk.htmtm-hannover.de (M.R.); Eva.Baumann@ijk.htmtm-hannover.de (E.B.)

**Keywords:** psychological distress, coping ability, genetic counseling, risk assessment, hereditary breast and ovarian cancer (HBOC)

## Abstract

Carriers of pathogenic variants causing hereditary breast and ovarian cancer (HBOC) are confronted with a high risk to develop malignancies early in life. The present study aimed to determine the type of psychological distress and coping ability in women with a suspicion of HBOC. In particular, we were interested if the self-assessed genetic risk had an influence on health concerns and coping ability. Using a questionnaire established by the German HBOC Consortium, we investigated 255 women with breast cancer and 161 healthy women before they were seen for genetic counseling. The group of healthy women was divided into groups of high and low self-assessed risk. In our study, healthy women with a high self-assessed risk stated the highest stress level and worries about their health and future. A quarter of the women requested psychological support. Overall, only few women (4–11%) stated that they did not feel able to cope with the genetic test result. More women (11–23%, highest values in the low-risk group) worried about the coping ability of relatives. The results of our exploratory study demonstrate that the women, who presented at the Department of Human Genetics, Hanover Medical School, Germany were aware of their genetic risk and had severe concerns about their future health, but still felt able to cope with the genetic test result.

## 1. Introduction

Breast and ovarian cancers are among the most common cancers in women in the developed world [1]. Approximately 5–10% of all breast cancers and up to 25% of all ovarian cancers have a genetic predisposition. Up to now, 25% of all hereditary breast and ovarian cancers (HBOCs) can be explained by the highly penetrant risk genes *BRCA1* and *BRCA2* and up to 15% by other HBOC risk genes (e.g., *RAD51C*, *RAD51D*, *ATM*, *CHEK2*, *BRIP1*, *PALB2*, *BARD1*, *RECQL*, *TP53*, and *CDH1*) [1,2]. Women carrying a pathogenic variant are at high risk of developing breast or ovarian cancer. The lifetime risks for carriers of a *BRCA1*-pathogenic variant range from 65% to 79% for breast cancer and from 36% to 53% for ovarian cancer [3]. In families with HBOC, usually several close relatives disease, often early in life, or even die from breast and ovarian cancer [4]. These individual and family characteristics can be used to assess the personal risk to carry a pathogenic variant, the need for intensified screening, and a potential offer of risk-reducing surgery. Genetic counseling is the process of identifying and counseling persons at risk for familial or inherited cancer diseases and is mandatory before genetic testing in Germany as declared by the German Genetic Diagnostics Act. To identify persons at risk, these women are offered genetic testing of risk genes. Carriers of pathogenic variants of *BRCA1* and *BRCA2* are referred to an intensified surveillance, including clinical examination, ultrasound, mammography and MRI scan in order to detect breast cancer at an early stage. Furthermore, prophylactic surgery, i.e., salpingo-oophorectomy, is recommended to them [5]. The diagnosis of cancer and its treatment can lead to cancer-related distress or different mental disorders such as depression and anxiety [6,7]. Further, being a carrier of a *BRCA1-* or *BRCA2*-pathogenic variant can be severely distressing as it means living with a lifelong risk of developing cancer [8]. There has been only limited evidence of adverse psychosocial outcomes following genetic testing to assess cancer risk in longitudinal studies [9]. Cancer-affected carriers have shown no significant increase in psychological distress in the long term after disclosure of a positive test [10].

Psychological distress is a broad term that commonly includes anxiety, stress, worry, panic, and fears [11]. In this study, we asked for specific fears, worries and stressors that may arise in women at high risk of hereditary breast and ovarian cancer before undergoing genetic counseling. Do women show an increased level of psychological distress before undergoing genetic counseling to investigate their risk for HBOC? Within this group of women: Do women with breast cancer have a higher level of psychological distress than healthy women? This leads to the next question: Does a high versus a low self-assessed genetic risk for HBOC in healthy women have an effect on the (1) presence of mental illnesses, concentration or sleep disturbances, (2) stress exposure, (3) fears to cope with the results of the genetic testing and challenges to communicate the results within their families due to relatives’ lack of coping abilities, and (4) request for psychological counseling. Further, we aimed to explore differences between women with and without children regarding different forms of problems with genetic testing regarding HBOC.

## 2. Materials and Methods

### 2.1. Subjects

Initial inclusion criteria covered women between the age of 18 and 80 years with suspected HBOC, who presented at the Department of Human Genetics, Hanover Medical School for genetic counseling and genetic testing. Exclusion criteria were insufficient German language skills. Between 2012 and 2017, 416 women were included in the study. The suspicion of HBOC was defined according to the criteria of the German Consortium of HBOC (2012–2017):

(1) For Index patients, the family had to fulfill the following criteria:

⁃ three women with breast cancer or
⁃ two women with breast cancer, one of them under the age of 51 years or
⁃ one woman with breast cancer and one woman with ovarian cancer or
⁃ two women with ovarian cancer or
⁃ one woman with breast cancer and one man with breast cancer or
⁃ one woman with ovarian cancer and one man with breast cancer or
⁃ one woman with breast cancer under the age of 36 years or
⁃ one woman with bilateral breast cancer and first diagnosis with 50 years or before or
⁃ one woman with breast and ovarian cancer.

(2) Women, in whose family no Index patients were alive or present and risk calculation of heterozygote risk (HZR) and breast cancer lifetime risk (LZR) using pedigree risk calculation program CYRILLIC 2.1 was HZR >= 20% and/or LZR => 30%

(3) Women, in whose family a pathogenic variant had been detected before.

For women, in whose family a pathogenic variant had been diagnosed before (3) a predictive test was offered. For index patients (1) and healthy women (2) fulfilling the criteria given above a diagnostic test was offered. The following genes were analyzed in 2012–2016: *BRCA1*, *BRCA2*, *CHEK2*, in case of additional ovarian cancer; additionally, *RAD51C*. In 2016 and the following years, the gene panel was continually expanded for several more genes including *PALB2*, *ATM*, *CDH1*, *NBN*, *RAD51D*, and *TP53*. All women provided written consent, approved by the Ethics Committee of Hannover Medical School (No. 4121, 1 February 2010, updated 9 March 2015). Sociodemographic variables, such as age, parenthood, and family history of cancer, were pseudonymized.

### 2.2. Measures 

The questionnaire was developed and validated by psychologists and other members of the German HBOC Consortium [12]. The questionnaire assessed age and age of first breast cancer diagnosis if respondents suffered from breast cancer. Further, respondents were asked about their family situation (having children, number of children, age of the youngest child) and their breast and ovarian cancer family history (if the mother and/or at least one sister or other relatives were diagnosed with breast and ovarian cancer and if so, at what age; if the mother and/or sister died from breast or ovarian cancer; and the number of other relatives diagnosed with breast or ovarian cancer or other cancer types). Genetic risk perception was measured with one item (“How do you currently assess your personal hereditary risk (from 0 to 100)”) on a scale from 0 to 100 with higher numbers indicating higher perceived risk. A closed-ended questionnaire (‘yes’ or ‘no’) assessed different indicators of psychological distress: Items included suffering from concentration or memory dysfunctions and suffering from insomnia. We asked for the presence of mental illnesses by self-assessment (‘yes’ or ‘no’). Provided there was a self-reported mental illness, the women were asked to specify their mental illness in an open-ended question. Since the assessment of mental illnesses is self-reported, no information is available whether reported mental illnesses were clinically diagnosed or not. In addition, four items asked if the respondents are at the moment or were in the last two years exposed to stress caused by different factors (partnership problems, problems within the family, job-related problems, financial problems). In two further items, respondents were asked to state if they assess themselves as generally rather anxious and pessimistic (‘yes’ or ‘no’). Four items (‘yes’ or ‘no’) measured potential problems with genetic testing due to worries about the future, due to health concerns, due to a lack of coping abilities, due to relatives’ lack of coping abilities. Finally, respondents were asked to state if they request psychological counseling either for decision support or in helping to cope (‘yes’ or ‘no’). All translated items are given in the appendix of the supplementary material (Table S1).

### 2.3. Data Analysis 

The sample was divided into three groups by first separating healthy women and women suffering from breast cancer. Healthy women were asked to estimate their subjective numeric genetic risks. Healthy women were then divided into two groups based on their self-assessed genetic risk. A median split (median = 50) was performed to divide healthy women into two groups of low (50 or lower) vs. high (higher than 50) self-assessed risk. Besides descriptive analyses of the sample using absolute and relative frequencies for categorical data and means (*M*) and standard deviations (*SD*) for numeric data, differences between the three groups and between respondents with and without children were assessed using Pearson’s chi-square tests. In the case of expected cell frequencies below 5, a Fisher’s exact test was performed. The data is characterized by several non-responses (valid answers in different questions ranging from 29% to 100%). Thus, all reported results are based on those respondents who answered the respective question and are therefore presented as valid percentages. In the tables, the percentage of valid answers for each question is indicated and total percentages as well as valid percentages are reported. In chi-square tests, cases with missing values were removed. All data were analyzed using IBM SPSS Statistics for Windows (v. 27, Armonk, NY, USA: IBM Corp.).

## 3. Results

### 3.1. Sample Characteristics

**Figure 1:**
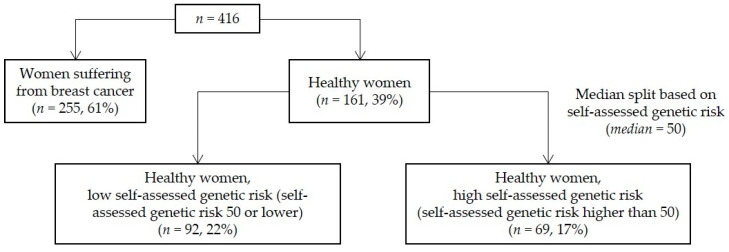
Overview of the sample and the three groups under investigation.

Breast cancer was diagnosed previously in 255 women (61%), the remaining 161 women are referred to as healthy women (39%). None of the investigated women was suffering from ovarian cancer. The healthy respondents were further divided based on their self-assessed genetic risk, which resulted in three groups in total: (1) women suffering from breast cancer (*n* = 255, 61%), (2) healthy women with low self-assessed genetic risk (*n* = 92, 22%), and (3) healthy women with high self-assessed genetic risk (*n* = 69, 17%). Figure 1 gives an overview of the sample and the three groups.

Table 1 shows the sample characteristics within the three different groups. A total of 161 women (39%) with suspected HBOC were healthy. In the first group there were 92 women (22%) with low self-assessed risk. The mean age was 39 and 57% had children. On average, women in this group had one child at the age of 14. The family history of these women revealed that in 60% of the cases the mother had been diagnosed with breast cancer, who had on average been 48 years old at the time of diagnosis. Further, 53% (32% of all respondents in this group) of the respondents who answered this question indicated that their mother had died from breast cancer, while 36% (22% of all respondents in this group) of respondents who answered this question indicated that at least one of their sisters was diagnosed with breast cancer, on average at the age of 40, and 14% (3% of all respondents in this group) of the respondents who answered this question stated that at least one of their sisters died from breast cancer. In the first group, on average 1.96 of the respondents’ other relatives (besides their mothers and sisters) were diagnosed with breast cancer, 0.62 were diagnosed with ovarian cancer, and 2.54 were diagnosed with other cancer types.

Sixty-nine (17%) of the women belonged to the second group, healthy women with high self-assessed risk. The mean age was 37 years, which was comparable to healthy women with low self-assessed risk. Two thirds of the women had children (67%): On average, women in this group had one child at the age of 9. The family history was comparable to the first group. In 64% of the cases the mother was diagnosed with breast cancer, on average at the age of 47, and 55% (35% of all respondents in this group) of the respondents who answered the question stated that their mother had died from breast cancer, and 34% (21% of all respondents in this group) of respondents who answered this question indicated that at least one of their sisters was diagnosed with breast cancer, on average at the age of 40. Moreover, 21% (4% of all respondents in this group) of the respondents who answered this question stated that at least one of their sisters died from breast cancer. In this group, most other relatives were diagnosed with cancer. On average, 1.63 of the respondents’ other relatives were diagnosed with breast cancer, 0.46 were diagnosed with ovarian cancer, and 2.41 were diagnosed with other cancer types.

The third group included 255 women who were previously diagnosed with breast cancer (61%). In this group, the mean age was with 51 years higher compared to the mean age in healthy women (39 and 37 years old). Likewise, the current age of the youngest child, was higher (22 years compared to 14 and nine years). Most women (80%) had two children. Compared to the healthy women, fewer women had a mother who had been diagnosed with breast cancer (29%) and the age of the mother at the time of diagnosis had been higher (58 years). Fewer mothers (59% of the respondents who answered this question; 17% of all respondents in this group) had died from breast cancer than compared to the healthy women, while 25% (16% of all respondents in this group) of respondents with breast cancer who answered this question indicated that at least one of their sisters was diagnosed with breast cancer, on average at the age of 53, and 23% (10% of all respondents in this group) of the respondents who answered this question stated that at least one of their sisters died from breast cancer. In the third group, on average, 1.12 of the respondents’ other relatives were diagnosed with breast cancer, 0.30 were diagnosed with ovarian cancer, and 2.51 were diagnosed with other cancer types. Thus, on average, fewer other relatives were diagnosed with breast or ovarian cancer than in the other two groups.

### 3.2. Most Healthy Women Considered Their Genetic Risk to Be High

**Figure 2:**
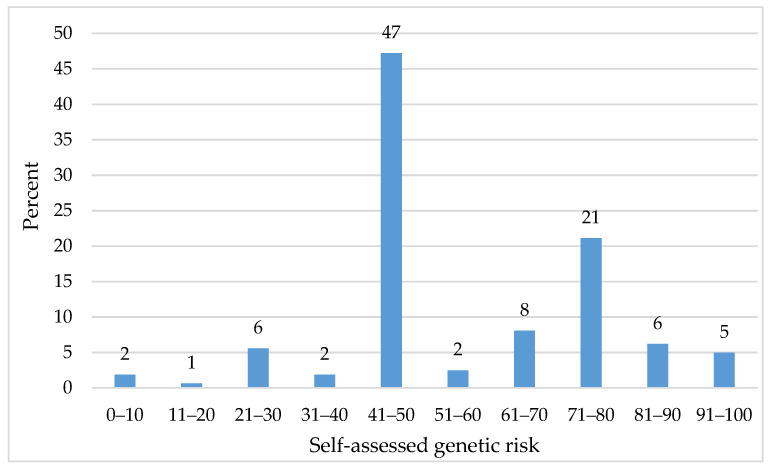
Self-assessed genetic risk of healthy women (*n* = 161). Note: The *y*-axis maximum was truncated.

In order to analyze the differences of psychological distress depending on cancer status and risk perception in women with suspected HBOC, we asked the participating women to estimate their subjective numeric genetic risks (Figure 2).

Across all healthy women under investigation, the self-assessed risk perception was *M* = 60.34 (*SD* = 20.00; min = 0; max = 100). Almost half of the women reported their perceived risk with exactly 50 (47%), 9% with 11–40 and 38% with 51–99. Two percent estimated that they have no genetic risk (self-assessed risk: 0) and four percent thought they will definitely develop HBOC (self-assessed risk: 100).

### 3.3. Women with Breast Cancer Showed the Highest Level of Suffering from Concentration and Memory Disfunctions as Well as Insomnia

**Figure 3:**
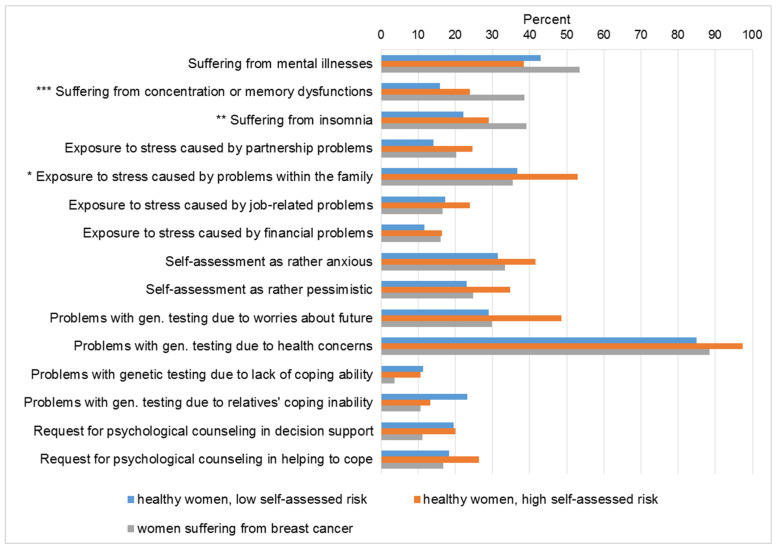
Psychological distress in women with suspected hereditary breast and ovarian cancer before genetic counseling. Total *n* = 416. Percentages are presented based on the respondents who answered the questions (valid answers ranging from 33–98%). Self-assessed risk 0–50: healthy women, low self-assessed risk; self-assessed risk 51–100: healthy women, high self-assessed risk. * *p* ≤ 0.05; ** *p* ≤ 0.01; *** *p* ≤ 0.001.

In this study, we asked for specific fears, worries and stressors to analyze psychological distress that may arise before women at high risk of hereditary breast and ovarian cancer come to the Department of Human Genetics for genetic counseling. First, we asked if the women were suffering from mental illnesses. Between 38% and 54% affirmed this question (Figure 3). There was no significant difference between the three groups. Asked to specify the mental illness in an open-ended question, 58% of the women reported depression, 15% reported anxiety and panic disorders, followed by stress and adjustment disorders (7%), burnout (3%), eating disorders (2%), and personality disorders (2%). When asked about different types of psychological distress such as concentration, memory and sleep disturbances, there was a significant difference between the three groups (concentration or memory dysfunctions: χ² (2, *n* = 391) = 17.28; *Cramer-V* = 0.21; *p* ≤ 0.001; insomnia: χ² (2, *n* = 407) = 9.25; *Cramer-V* = 0.15; *p* ≤ 0.01). Significantly more women with breast cancer were suffering from these disorders. Women who considered their own risk to be low were the least affected by concentration, memory, and sleep disorders.

### 3.4. Healthy Women with a High Self-Assessed Risk Showed the Highest Level of Stress Exposure and Were More Inclined to Request Psychological Support

In this study, women with a high self-assessed genetic risk tended to be more exposed to stress caused by problems within family or their partnership and also by job-related problems compared to women with low self-assessed genetic risk and women suffering from breast cancer. The three groups differed significantly regarding stress exposure by problems within the family (χ² (2, *n* = 401) = 7.10; *Cramer-V* = 0.13; *p* ≤ 0.05), which played a great role for all women (35–53%), whereas stress exposure by financial problems was the least frequently mentioned cause of stress (12–16%). Particularly, women with a high self-assessed genetic risk were more likely to rate themselves as anxious and pessimistic than women with a low self-assessed risk or women suffering from breast cancer, although this difference was not statistically significant. There were also no significant differences between the three groups regarding different problems with genetic testing and requests for psychological counseling. Nevertheless, descriptively, our results showed that women with a high self-assessed genetic risk worried about the future more frequently compared to the other groups (48% versus 28% and 29%). Health concerns were the most prominent among all participants (85–97%). Healthy women were only rarely concerned about a possible lack of coping ability after genetic testing (11–12%). Women suffering from breast cancer were even less concerned about a possible lack of coping ability (3%) or due to their relative’s lack of coping abilities (11%). Healthy women with a high self-assessed risk worried less about a possible lack of coping ability of relatives than women with a low self-assessed risk (13% vs. 23%). Psychological counseling for decision support was requested by healthy women the most (19% and 20% versus 12%). Some women (16–26%) wished to receive psychological counseling in order to cope with the test result.

### 3.5. Women without Children Worried Significantly More about the Future Than Women with Children

In order to examine the problems with genetic testing in more detail, we analyzed differences between women with and without children (Table 2). Results showed only a small, but significant difference regarding the respondent’s feelings about the future (χ² (1, *n* = 170) = 11.02; φ = 0.26; *p* ≤ 0.001). While 51% (25% of all respondents without children) of respondents without children who answered this question reported problems with genetic testing because of worries about the future, only 25% (10% of all respondents with children) of women with children who answered this question indicated such worries.

## 4. Discussion

In this study, we analyzed the psychological distress of women with suspected HBOC before they presented for genetic counseling and testing. Besides analyzing the difference in psychological distress of women with or without breast cancer, we were interested if psychological distress was associated with self-assessed genetic risk. Most women considered their genetic risk to be high. In a study of Rutherford et al. [13], 84% of women with a family history of breast cancer have overestimated their personal lifetime risk of developing breast cancer. This suggests consistency with our results. However, in contrast to Rutherford et al. [13], we did not assess the respondents’ risk perception of developing breast cancer but their genetic risk perception. Since the women in our sample only received genetic counseling and testing because of suspected HBOC, their genetic risk perception–although high–seems rather realistic. Younger women have been demonstrated to have heightened and inaccurate perception of breast cancer risk compared to older women [14]. Furthermore, risk perception has been shown to be associated with personal experience of cancer within the family [15]. This could have influenced the genetic risk perception of the women in our study–especially, since respondents were confronted with the cancer history in their family while filling out the surveys necessary for genetic counseling. In the following sections, we discuss the influence of suffering from breast cancer and of risk perception on psychological distress and stress exposure, challenges with genetic testing, and request for psychological counseling.

Focusing on different types of psychological distress and stress exposure, we observed that a large proportion of women reported suffering from depression. It has been shown that especially women with breast cancer who undergo the process of genetic testing are confronted with breast cancer-related fear and have an elevated level of vulnerability compared to healthy individuals at increased risk for breast cancer [16]. The diagnosis of cancer and its treatment have been shown to lead to high level of cancer-related distress [7]. Even clinically relevant symptoms such as depression or anxiety have been mentioned to be elevated in this group [17]. In our study, though, we did not find significant differences regarding different types of psychological distress between healthy women and women diagnosed with breast cancer.

Women in our study showed a high level of stress exposure especially due to problems within their family. Women suffering from breast cancer have been reported to develop stress disorders or even posttraumatic events as a result of the breast cancer diagnosis [18]. However, in our study, healthy women with high self-assessed risk showed a significantly higher level of stress exposure due to problems within the family than women with breast cancer. Instead, women with breast cancer were suffering from concentration, memory and sleep disorders more often compared to healthy women. This could be due to the treatment the women had received (e.g., chemotherapy). Unfortunately, we had no information about the treatment women with breast cancer had undergone at the time of data collection.

Furthermore, we observed that women with a high self-assessed risk rated themselves as rather anxious and pessimistic and had concerns about their future. Women with an increased risk of developing breast cancer have displayed traumatic responses related to the genetic testing process similar to cancer patients [19]. We suppose that genetic counseling and genetic testing for HBOC reactivates the women’s experiences with cancer histories in their families and thus leads to an increased fear of a personal cancer diagnosis and a higher level of psychological distress. This seems to be particularly true for women with a high self-assessed risk.

Women in all analyzed groups reported worries about their health as a result from genetic testing. Notably, only a few women stated a lack of coping ability. Coping has been described as a mediator of the relationship between illness perception and distress in the Self-Regulatory Model of Illness Behavior [20]. Although coping has been described to be linked to anxiety and depression in breast cancer [21,22], only few of the women reported self-stated lack of coping ability. On the contrary, women with breast cancer as well as healthy women were more afraid of the lack of coping ability of their relatives. This is in line with several studies that have shown that disseminating information about detected pathogenic variants within families and across generations is complex and triggers many fears [23].

Although this study provides valuable insights into psychological distress of women with suspected HBOC, who presented for genetic counseling and testing at the Department of Human Genetics at the Hanover Medical School, the limitations of the study need to be considered. Our study is based on a small sample size and is not representative. Moreover, the data is characterized by several non-responses to some of the questions, which needs to be considered when interpreting the results. Since the results are mostly descriptive, missing values were not substituted to avoid distortion of the results. A median split always poses the problem of assigning respondents with medium values to one of the two groups (low vs. high) and, thus, making it impossible to reveal differences between this moderate group and the low and high groups. Therefore, our study only gives first insights into differences between women with relatively low- and high-risk perceptions regarding psychological distress. We agree that distinguishing between women with a low perceived risk vs. medium perceived risk vs. high perceived risk could yield further information and should be explored in future studies with larger sample sizes. Our findings hint to certain conclusions, but studies based on larger sample sizes are needed to further examine these first insights. In addition, the close-ended questions allowing only yes- or no-answers do not allow for in-depth conclusions on the subjects under investigation. Future research should collect more in-depth data to explore women’s rationale regarding different aspects in the context of genetic testing and counseling.

## 5. Conclusions

In conclusion, this study demonstrates that women undergoing genetic counseling to seek advice regarding hereditary breast and ovarian cancer, whether diseased from breast cancer or not, present with a variety of worries, particularly about their health and future. This should be taken into account and lead genetic counselors and doctors to actively addressing these personal concerns. For example, the counseling session could start with a short query to get an overview about the individual psychological distress of the counselees. Although most of the women stated that they felt able to cope with the test results, a quarter of the women requested psychological support. Therefore, psychologists familiar with psycho-oncology and genetic risk assessment should be integrated into multi-disciplinary teams of HBOC centers.

## Figures and Tables

**Table 1:** Sample characteristics.

	Healthy Women, Low Self-Assessed Risk (*n* = 92, 22%)		Healthy Women, High Self-Assessed Risk (*n* = 69, 17%)		Women Suffering from Breast Cancer (*n* = 255, 61%)	
	Valid Answers %	*n* (%) [Valid %]/ *M* (*SD*)	Valid Answers %	*n* (%) [Valid %]/ *M* (*SD*)	Valid Answers %	*n* (%) [Valid %]/ *M* (*SD*)
Age	100%	39.20 (*SD* = 11.26)	100%	36.99 (*SD* = 9.91)	100%	51.45 (*SD* = 11.67)
Age at first diagnosis of breast cancer					99%	47.57 (*SD* = 11.06)
Family situation						
Having children	100%	52 (57%) [57%]	100%	46 (67%) [67%]	100%	203 (80%) [80%]
Number of children	100%	0.98 (*SD* = 1.01)	100%	1.14 (*SD* = 1.03)	100%	1.55 (*SD* = 1.06)
Age of youngest child	55%	14.33 (*SD* = 9.58)	64%	9.30 (*SD* = 8.74)	78%	22.22 (*SD* = 12.46)
Breast cancer family history						
Mother diagnosed with breast cancer	99%	55 (60% [60%]	100%	44 (64%) [64%]	99%	75 (29%) [30%]
Mother’s age at first diagnosis of breast cancer	60%	48.35 (*SD* = 10.56)	61%	47.05 (*SD* = 13.69)	29%	57.80 (*SD* = 14.41)
Mother died from breast cancer	60%	29 (32%) [53%]	64%	24 (35%) [55%]	29%	44 (17%) [59%]
At least one sister diagnosed with breast cancer	61%	19 (21%) [34%]	61%	15 (22%) [36%]	64%	41 (16%) [25%]
Youngest affected sister’s age at first diagnosis of breast cancer	21%	40.32 (*SD* = 9.67)	22%	40.13 (*SD* = 8.68)	16%	52.57 (*SD* = 10.37)
At least one sister died from breast cancer	21%	4 (4%) [21%]	20%	2 (3%) [14%]	17%	10 (10%) [23%]
Number of other relatives diagnosed with breast cancer	100%	1.63 (*SD* = 1.40)	100%	1.96 (*SD* = 1.59)	99%	1.12 (*SD* = 1.23)
Number of other relatives diagnosed with ovarian cancer	100%	0.46 (*SD* = 0.79)	100%	0.62 (*SD* = 1.16)	99%	0.30 (*SD* = 0.61)
Number of other relatives diagnosed with other cancer types	100%	2.41 (*SD* = 2.24)	100%	2.54 (*SD* = 1.95)	99%	2.51 (*SD* = 2.02)

Note. *n* = 416. Genetic risk perception was measured on a scale from 0 to 100; a median split (median = 50) was performed to separate the healthy respondents in groups with low self-assessed risk (perceived risk ≤ 50) vs. high self-assessed risk (perceived risk > 50); total percentages as well as valid percentages based on those respondents who answered the respective question are reported to facilitate interpretation of the data, which is characterized by several non-responses.

**Table 2:** Comparison of women with or without children regarding different problems with genetic testing.

	Women without Children (*n* = 114, 28%)		Women with Children (*n* = 301, 73%)	
	Valid Answers%	*n* (%) [Valid %]	Valid Answers%	*n* (%) [Valid %]
Problems with genetic testing, because				
I cannot cope with the test result	39%		34%	
No		41 (36%) [93%]		95 (32%) [93%]
Yes		3 (3%) [7%]		7 (2%) [7%]
my relatives cannot cope with the test result	33%		32%	
No		33 (29%) [87%]		83 (28%) [86%]
Yes		5 (4%) [13%]		14 (5%) [14%]
I am afraid of the future ***	48%		38%	
No		27 (24%) [49%]		86 (29%) [75%]
Yes		28 (25%) [51%]		29 (10%) [25%]
I am concerned about my health	49%		42%	
No		3 (3%) [5%]		16 (5%) [13%]
Yes		53 (47%) [95%]		109 (36%) [87%]

*Note*. *n* = 415 (one missing answer regarding having children). Total percentages as well as valid percentages based on those respondents who answered the respective question are reported to facilitate interpretation of the data, which is characterized by several non-responses; chi-square test: *** *p* ≤ 0.001.

## Data Availability

All data can be made available upon request.

## Supplementary Materials

The following are available online at https://www.mdpi.com/article/10.3390/ijerph18084338/s1, Table S1: Measurements.
