# Supplementary material for: Psychological Distress and Coping Ability of Women at High Risk of Hereditary Breast and Ovarian Cancer before Undergoing Genetic Counseling—An Exploratory Study from Germany
Source: Int J Environ Res Public Health. 2021 Apr 19;18(8):4338. doi: 10.3390/ijerph18084338 (PMC8073852; doi:10.3390/ijerph18084338)
Supplement: Supplementary file 1 [file ijerph-18-04338-s001.zip › ijerph-1116433-supplementary.pdf]

International Journal of Environmental Research and Public Health

Table S1. Measurements (all items were translated verbatim from German)

**Genetic risk perception**	
"How do you currently assess your personal hereditary risk (from 0-100)"	0-100
**Self-assessment of suffering from mental illness**	
"Do you suffer from mental illness (e.g. depression)"	yes/no
"If yes: which?"	open ended
**Self-assessment of suffering from concentration or memory dysfunctions**	
"Do you suffer from concentration or memory problems?"	yes/no
**Self-assessment of suffering from insomnia**	
"Do you suffer from insomnia?"	yes/no
**Exposure to stress currently or in the last two years**	
"Are you currently or in the last two years burdened (stress) …	
…due to partnership problems (e.g., separation)?"	yes/no
…due to problems within the family (e.g., worries about the children)?"	yes/no
…due to job-related problems (e.g., bullying)?"	yes/no
…due to financial problems?"	yes/no
**Self-assessment as rather pessimistic and anxious**	
"How do you rate yourself?	
I am generally more pessimistic."	yes/no
I am generally rather anxious."	yes/no
**Problems with genetic testing**	
"Are you having problems with the genetic test?	
I cannot cope with the test result."	yes/no
My relatives cannot cope with the test result."	yes/no
I am afraid of the future (family planning etc.)."	yes/no
I am concerned about my health."	yes/no
**Request of psychological counselling**	
"Would you currently like to receive psychological counselling?	
…in decision support?"	yes/no
…in helping to cope?"	yes/no
